# Artificial intelligence-based image analysis can predict outcome in high-grade serous carcinoma via histology alone

**DOI:** 10.1038/s41598-021-98480-0

**Published:** 2021-09-27

**Authors:** Anna Ray Laury, Sami Blom, Tuomas Ropponen, Anni Virtanen, Olli Mikael Carpén

**Affiliations:** 1 Research Program in Systems Oncology, Research Programs Unit, Faculty of Medicine, University of Helsinki, Helsinki, Finland; 2 iCAN Digital Precision Cancer Medicine Flagship, University of Helsinki, Helsinki, Finland; 3 Department of Pathology, University of Helsinki and HUS Diagnostic Center, Helsinki University Hospital, Helsinki, Finland; 4 Aiforia Technologies Oy, Helsinki, Finland

**Keywords:** Translational research, Ovarian cancer

## Abstract

High-grade extrauterine serous carcinoma (HGSC) is an aggressive tumor with high rates of recurrence, frequent chemotherapy resistance, and overall 5-year survival of less than 50%. Beyond determining and confirming the diagnosis itself, pathologist review of histologic slides provides no prognostic or predictive information, which is in sharp contrast to almost all other carcinoma types. Deep-learning based image analysis has recently been able to predict outcome and/or identify morphology-based representations of underlying molecular alterations in other tumor types, such as colorectal carcinoma, lung carcinoma, breast carcinoma, and melanoma. Using a carefully stratified HGSC patient cohort consisting of women (n = 30) with similar presentations who experienced very different treatment responses (platinum free intervals of either ≤ 6 months or ≥ 18 months), we used whole slide images (WSI, n = 205) to train a convolutional neural network. The neural network was trained, in three steps, to identify morphologic regions (digital biomarkers) that are highly associating with one or the other treatment response group. We tested the classifier using a separate 22 slide test set, and 18/22 slides were correctly classified. We show that a neural network based approach can discriminate extremes in patient response to primary platinum-based chemotherapy with high sensitivity (73%) and specificity (91%). These proof-of-concept results are novel, because for the first time, prospective prognostic information is identified specifically within HGSC tumor morphology.

## Introduction

Ovarian cancer is the 5th leading cause of cancer death in women in the United States^1^ and other western countries; about three quarters of those deaths are due to high-grade serous carcinoma (HGSC)^2^. Extrauterine high-grade serous carcinomas (ovary, fallopian tube, peritoneum) are aggressive tumors with poor outcomes; overall 5 year survival is less than 50%^1,3^, and for women with advanced disease, about 25%. These tumors typically present at late stage (III–IV), and are remarkable for their genetic and morphologic heterogeneity^4^; they are also notable for their diversity in outcome. Well-described clinical factors affecting long term survival include younger age at diagnosis, lower stage, and optimal surgical debulking, however, even within these strata outcome is unpredictable^5^. Unlike many other cancer types, some patients (up to 15%) presenting with stage III–IV disease can survive for a decade or beyond^6,7^, suggesting significant underlying biologic differences within HGSC. Until recently, the standard treatment for these tumors had changed very little since the 1970s, consisting of debulking surgery and platinum-based chemotherapy, with very little improvement in survival. Since 2014, poly ADP ribose polymerase (PARP) inhibitors^8^ have been available, which work by exploiting DNA repair vulnerabilities in some patients, including those with BRCA mutations. Despite the ever-increasing body of knowledge on the role of BRCA mutations and homologous recombination (HR) deficiencies in these high grade tumors, treatment response remains unpredictable and disease progression is poorly understood, even in patients presenting with advanced disease^9,10^.

We know that in addition to PARP inhibitor sensitivity, women with BRCA mutations and HR deficiency are more likely to have a good response to standard therapy^11–14^, though overall, treatment response is not entirely predictable even if the underlying mutation(s) are known. Further, HR deficiency is only present in about half of tumors, and testing for HRD remains a challenge^15,16^; additional tools for treatment response prediction are needed. While the relevance of the progression free interval (PFI) as an indicator is debatable and somewhat arbitrary, women who relapse in < 6 months are considered platinum resistant, while those with a PFI of > 12 months are considered platinum sensitive. Only a quarter of women have a PFI of > 18 months, which has been reported as a conditional prognostic factor for long term survival^17^; a small subset of patients experience prolonged, long term survival (≥ 7 years), but very little in the way of additional prognostic information is known about these women^5,18^.

The variability in morphology of HGSC is notable both inter- and intratumorally; unlike many other cancer types a clinically useful correlation between morphology and outcome has been difficult to define. Within BRCA mutations and HR deficiency certain morphologic patterns have been identified more frequently, however, these sometimes contradictory findings have not been sufficiently discriminatory for clinical use^19–22^.

The past 10 years have also brought about significant advances in digital pathology; whole slide scanning has become faster, computational power has increased, and data storage has become cheaper. These developments have made large-scale slide scanning and image analysis a reality, and allowed the development of machine learning tools to address biological questions via histology and tumor morphology. A variety of methods have rapidly begun to prove their potential clinical utility. Machine algorithms can perform as well as expert pathologists in some types of cancer diagnosis^23,24^, offer additional precision at error-prone identification tasks such as metastasis detection^25^, and perhaps most importantly, have the potential to add novel information. There is already a growing body of evidence showing that artificial intelligence-based image analysis can identify morphologic features in tumors which reflect underlying genetic differences^26,27^, and can aid in prognostication^28,29^.

Thus far, no similar studies have used whole slide images (WSI) to evaluate the utility of artificial intelligence-based image analysis in high grade extrauterine serous carcinoma. The premise of this project is that there is an intrinsic difference in HGSC tumors that are refractory to therapy from the outset, and those which take much longer to recur. Further, we propose that some indication of this underlying difference is detectable in the tumor morphology by artificial intelligence, and can be used to prospectively identify these two groups of patients, a task not currently possible prospectively. We hypothesize that with the help of a very well curated patient series reflecting the extremes of treatment response, we can train a neural network to differentiate between good outcome and poor outcome tumors in WSI. The purpose of this work was to determine if a weakly supervised convolutional neural network can accurately classify high grade serous carcinoma into outcome groups using tumor morphology alone.

## Methods

### Training and testing cohorts

An initial group of more than 1000 patients diagnosed with high-grade extrauterine serous carcinoma and treated at HUS Helsinki University Hospital between 1982 and 2013 was identified using diagnostic code searches. The following clinical data were recorded; age, stage, residual disease after surgery (R status), treatment protocol, PFI, recurrence status and type, vital status.

The study protocol and use of all material was approved by The Ethics Committee of the Hospital District of Helsinki and Uusimaa (HUS: HUS359/2017); all methods were carried out in accordance with the relevant guidelines and regulations. The clinical material was collected under the auspices of Helsinki Biobank. At the time of this work, Finnish biobank legislation (Biobank Act 688/2012, https://www.finlex.fi/en/laki/kaannokset/2012/en20120688.pdf) provided a lawful basis for the use of biobanked samples and data for scientific research without project-specific consent from the patients involved. For retrospective studies of remnant tissue (and deceased patients), specific consent was not required by Finnish law for samples obtained prior to 2013. Sample donors always have the right to withdraw from participation.

From this cohort, we selected patients with stage III-IV disease at presentation, who underwent primary cytoreductive surgery (any R status), and at least 6 cycles of adjuvant platinum-based chemotherapy. From this subset, we selected two distinct groups of women for inclusion in our training set; those with evidence of biochemical remission/response at some point during treatment (defined as CA-125 < 35 IU/ml) and either 1) extended progression free survival (≥ 18 months, n = 13) (PFI-L) or 2) a very short time to progression (≤ 6 months, n = 17)(PFI-S). Time to progression was defined as the platinum free interval (PFI); the time between the last chemotherapy cycle to the first recurrence. Recurrence was defined as any diagnosis of recurrent or progressive disease; laboratory, clinical, or radiologic. Extensive clinical data, including precise treatment and outcome information, has been verified. For each case, archived H&E slides were reviewed, the histologic diagnosis was verified, and confirmatory immunohistochemical stains (WT-1, p53) were evaluated. All available H&E slides of adnexal tumor masses from the primary debulking surgery were included in the training set (n = 205, 2–13 per woman).

The validation test cohort (n = 22) consisted of a separate group of women with the same selection criteria as the training cohort; only one slide from each woman was included in the validation test set. The slide with the greatest amount of well-preserved tumor tissue was selected as the representative slide.

### Morphologic review

The training set (n = 205 from 30 patients) and test set (n = 22) WSI were evaluated by a pathologist as described previously^19,20^ for classic papillary features versus solid, endometrioid, and transitional (SET) patterns or ambiguous morphology. All slides were from adnexal masses. The predominant morphologic pattern (> 50%) was recorded as either classic or non-classic (including SET, ambiguous). Uniform tumor morphology was also noted, and the presence of any classic papillary architecture was recorded.

### Slide preparation

All slides were prepared from archival formalin fixed paraffin embedded (FFPE) tissue blocks, and stained with hematoxylin & eosin (H&E) to create the whole slide images (WSI). Both archival and recut slides were used, depending on the condition of the original slide; if the original slide was faded or damaged, then recuts were obtained. The slides were digitized using a whole slide scanner; either Pannoramic SCAN 150 (3DHistech, Ltd., Budapest, Hungary) with an image resolution of 0.22 μm/pixel or a Pannoramic SCAN 250 with an image resolution of 0.24 μm/pixel, and uploaded to Aiforia’s cloud-based platform (Aiforia Technologies, Helsinki, Finland).

### Neural network workflow and training

**Figure 1:**
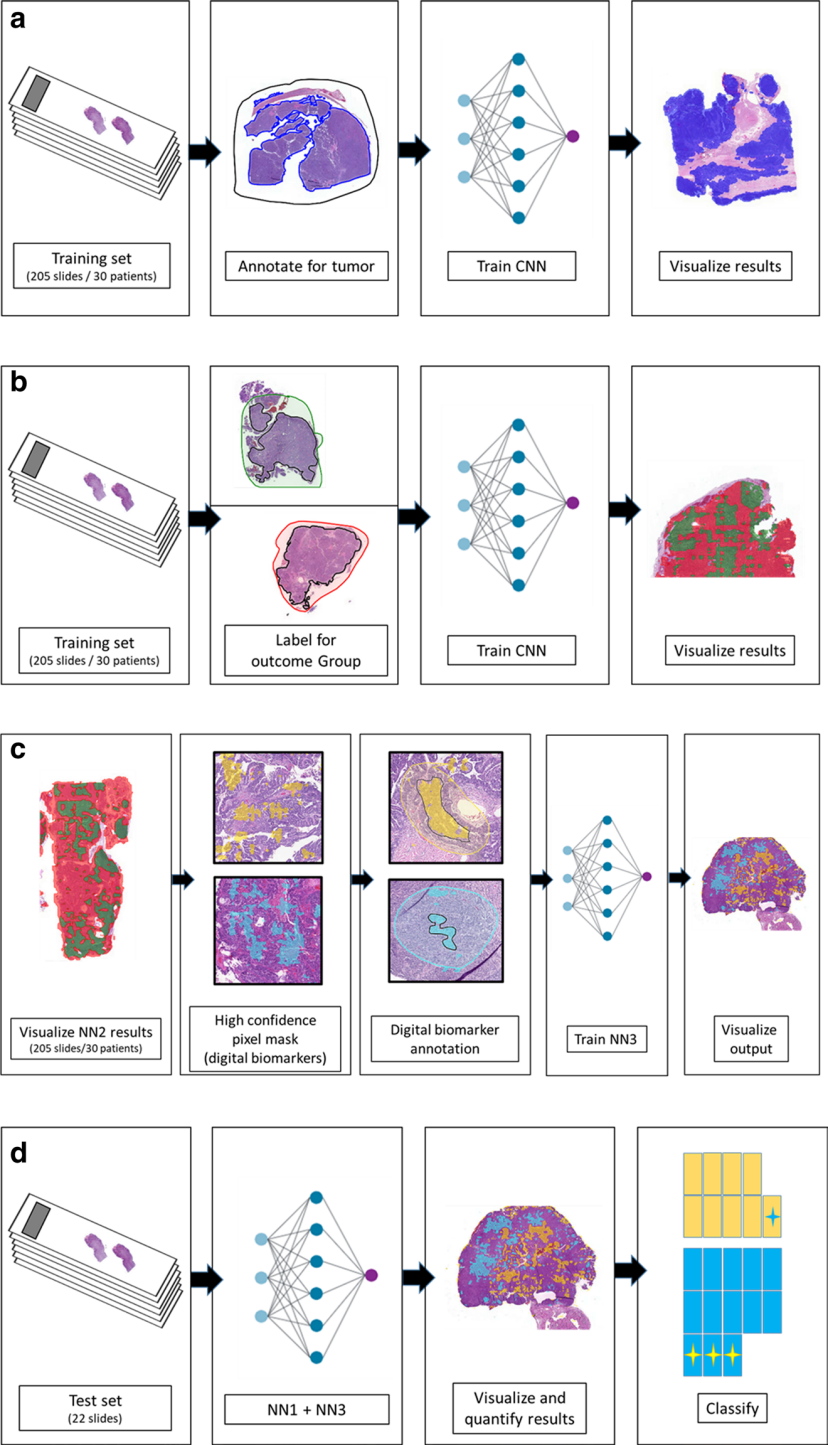
Neural network training. (**a**) Neural Net 1. Supervised learning of tumor segmentation. Manual semantic segmentation annotations are performed, and the neural network learns tumor segmentation. (**b**) Neural Net 2. Weakly supervised learning, using patient outcome group as the label. Tumor annotations from Neural Net 1 are relabeled as PFI-S or PFI-L, and the neural network learns tumor features associating with the two outcome groups. (**c**) Neural Net 3. Supervised learning based on annotations of digital biomarkers. The results of Neural Net 2 are visualized and confidence filtered. Features within the high-confidence masks (digital biomarkers) are reviewed and new semantic segmentation annotations of these regions are performed. The neural net learns tumor features that are strongly associating with outcome group. (**d**) Combined Inference Pipeline. Neural Net 1 and Neural Net 3 were combined in a single inference pipeline. The neural nets are applied to the validation test set; output is visualized and classified. Yellow slides represent WSI classified as PFI-S by the neural network, with the blue star indicating the misclassified WSI. Blue slides represent WSI classified as PFI-L by the neural network; those with yellow stars represent misclassified slides.

All tumor WSI were uploaded to Aiforia’s commercially available cloud-based platform, and the final neural network was trained in three steps (Fig. 1a–c) and applied to the test set (Fig. 1d). Training parameters are presented in Supplementary Table S1 online. The logical workflow proceeded as follows. First, a human (pathologist; ARL) shows a neural network where tumors are located within WSI. Then, the neural network shows the human which features in the WSI associate with patient outcome group (PFI-S and PFI-L). The human reviews the neural network’s suggestions, focusing on regions that are high-confidence for association with one or the other outcome group, and then tells the neural net to refine the interpretation of outcome-associated features based on the human’s review.

#### Neural network training

*Step 1* Neural network 1 (NN1), supervised learning of tumor segmentation. A semantic segmentation convolutional neural network was trained using hard labels for tumor regions. Manual pixel-level annotations were performed by a pathologist (ARL). Gross tumor regions were annotated in order to exclude background/benign tissue, artifacts (eg folded tissue), whitespace, and extensive necrosis. The network was trained with a 200 µm field of view.

*Step 2* Neural network 2 (NN2), weakly supervised learning using patient outcome as the label in tumor segments. The hard labels for tumor segments of the NN1 were relabeled based on patient outcome group (PFI-S, PFI-L). A convolutional neural network was trained to associate tumor features with the outcome group. The network was trained with a 500 µm field of view.

*Step 3* Neural network 3 (NN3), supervised learning based on hard labels for digital biomarkers. The output of NN2 was filtered based on pixel-level confidence (for association with one or the other outcome group). A digital biomarker is defined as a region of tumor identified by NN2 as being highly associated with one or the other PFI group. A mask showing only the high-confidence pixel regions (digital biomarkers) was reviewed by a pathologist (ARL), who curated (via manual annotation) a data set for training the new neural network. Regions identified by the high-confidence mask were annotated; annotations were focused viable tumor tissue and contiguous foci at least 200 µm in size. Regions of necrosis, stroma, tissue artifact, and blur were excluded. The neural network was trained with a 200 µm field of view, in replicate with identical training parameters (see Supplementary Table S1). The neural network learned tumor features strongly associating with the outcome group.

*Step 4* NN1 and NN3 were combined in a single inference pipeline in order to run final inference with output for semantic segmentation of tumor (NN1) and percentage of outcome classes within the tumor region (also semantic segmentation). NN1 and NN3 are not integrated at the network level; NN3 inference results are filtered by NN1 inference results.

*Step 5* Validation set testing. The combined inference pipeline was applied to the 22 slide validation test set. The output was visualized, and the WSI were classified according to rank order of the relative percent area of digital biomarkers identified within the tumors.

### Statistical analysis

Descriptive statistics were calculated in Microsoft Excel (2016) and via the online MedCalc statistical calculator (https://www.medcalc.org/calc/diagnostic_test.php).

## Results

### Cohort clinicopathologic features summary

The training set consisted of WSI (n = 205) from 30 women; 17 in the group PFI-S (WSI n = 105) and 13 in PFI-L (WSI n = 100). The validation test set consisted of 22 WSI from 22 women, 11 in the PFI-S group and 11 in PFI-L. The clinicopathologic features of each group are presented in Table 1.

**Table 1:** Clinicopathologic features summary.

	Training set		Validation test set	
	Short PFI (n = 17)	Long PFI (n = 13)	Short PFI (n = 11)	Long PFI (n = 11)
**Age at diagnosis (years)**				
Mean (range)	63.6 (54–75)	64.9 (51–73)	61.6 (43–78)	61.1 (50–73)
≥ 64	7	8	4	5
< 64	10	5	7	6
**FIGO stage**				
IIIB	1	2	0	4
IIIC	12	10	10	7
IVB	4	1	1	0
**R status after PDS**				
R0	1	3	0	2
R1	0	2	0	2
R2	16	8	11	7
**PFI (months)**				
Mean (range)	2.8 (0–5)	46.9 (19–149)	3.6 (0–6)	41.4 (18–87)
**Year of diagnosis (range)**	2006–2012	2007–2013	2006–2011	2006–2013
**Number of slides**	105	100	11	11
Mean per tumor (range)	6.2 (1–13)	7.7 (2–12)	1	1

### Morphologic review

Ten of the 30 training set cases were noted to have a predominantly papillary architecture (Table 2), relatively evenly balanced between the PFI-S (6/17, 35%) and PFI-L (4/13, 30%) outcome groups. 4 tumors were notable for their uniform morphology, all of which were the non-classic SET/ambiguous type. Only 6 tumors lacked any identifiable classic papillary architecture, and 4 of these represent the tumors with uniform morphology noted above. Findings were similar for the test set cases (Table 2), despite utilizing only one slide per tumor, though the PFI-S group had one case with uniform classic-type architecture.

**Table 2:** Morphologic classification of the training and testing sets.

Tumor morphology	Training set		Test set	
	PFI-S (n = 17)	PFI-L (n = 13)	PFI-S (n = 11)	PFI-L (n = 11)
Classic papillary (uniform)	6 (0)	4 (0)	3(1)	2(0)
Non-classic (uniform)	11 (2)	9 (2)	8(1)	9(1)
Classic foci, any amount	14	10	9	10

### Prognosticator/classifier

**Figure 2:**
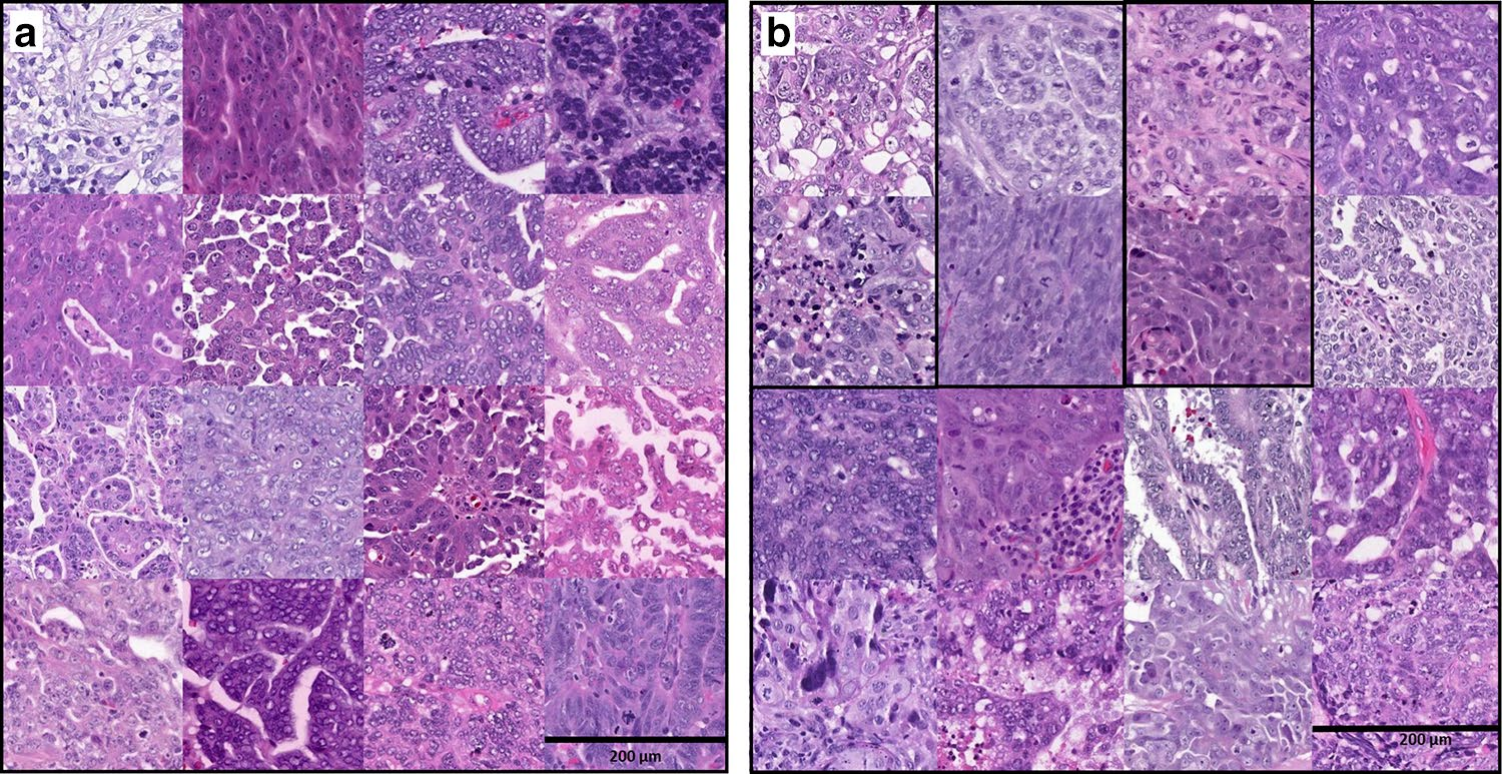
(**a**) Representative digital biomarkers for short PFI. Each tile (970 × 996px) is from a separate training set WSI and separate tumor. Each tile represents a region identified by NN2 as being highly associated with PFI-S, and that was annotated for inclusion in NN3 training. (**b**) Representative digital biomarkers for long PFI. Each tile (970 × 996px) is from a separate training set WSI; tiles from the same tumor are indicated within boxes. Each tile represents a region identified by NN2 as being highly associated with PFI-L, and that was annotated for inclusion in NN3 training.

We trained the classifier initially on 205 WSI of adnexal tumors from 30 women (Fig. 1a,b); this was followed by annotations of digital biomarkers (Fig. 1c; 178 WSI from the 30 women), which were used to train a new, final, neural network in replicate. In the 22 WSI validation test set, all slides had pixel areas identified as digital biomarkers (high-confidence pixel areas) for short PFI, and all but one slide had at least focal digital biomarker regions for long PFI, though the presence of PFI-L digital biomarker findings was scarce overall (Table 3). Representative digital biomarkers are shown in Fig. 2.

**Table 3:** Results and classification of validation test set replicates.

Replicate 1						Replicate 2					
Test set slide	Long- DBM area %	Short- DBM area %	Short/Long ratio	Outcome	AI prediction	Test set slide	Long-DBM area %	Short-DBM area %	Short/Long ratio	Outcome	AI prediction
T13	9.4	4.2	0.4	Long	Long	*T07*	*11.2*	*4.1*	*0.4*	*Short*	*Long*
T15	7.0	3.4	0.5	Long	Long	T15	15.8	7.4	0.5	Long	Long
T17	2.3	1.8	0.8	Long	Long	T16	16.2	9.7	0.6	Long	Long
T16	9.3	9.5	1.0	Long	Long	T13	8.1	7.9	1.0	Long	Long
*T07*	*5.4*	*7.0*	*1.3*	*Short*	*Long*	T17	6.5	8.6	1.3	Long	Long
T12	4.1	11.4	2.7	Long	Long	T14	6.2	17.0	2.8	Long	Long
*T08*	*3.6*	*16.0*	*4.4*	*Short*	*Long*	T12	8.0	28.8	3.6	Long	Long
T14	4.2	28.7	6.8	Long	Long	T20	4.5	18.5	4.1	Long	Long
*T02*	*1.1*	*12.6*	*11.4*	*Short*	*Long*	*T08*	*5.6*	*24.4*	*4.4*	*Short*	*Long*
T20	1.2	19.6	16.5	Long	Long	*T02*	*2.4*	*16.3*	*6.9*	*Short*	*Long*
T18	0.9	16.9	18.0	Long	Long	T19	1.2	22.0	18.7	Long	Long
T04	0.6	23.0	35.9	Short	Short	T18	1.5	38.2	25.1	Long	Long
*T19*	*0.3*	*11.4*	*36.4*	*Long*	*Short*	T21	1.9	49.6	25.7	Long	Long
*T22*	*0.3*	*10.4*	*37.4*	*Long*	*Short*	T03	2.2	66.5	30.2	Short	Short
T03	1.2	57.5	49.6	Short	Short	T09	0.2	30.2	124.7	Short	Short
*T21*	*0.2*	*25.9*	*104.6*	*Long*	*Short*	T11	0.5	78.2	146.5	Short	Short
T01	0.4	72.9	166.6	Short	Short	T06	0.4	63.8	181.0	Short	Short
T09	0.1	18.3	198.3	Short	Short	T04	0.3	48.9	186.3	Short	Short
T06	0.1	36.4	347.1	Short	Short	*T22*	*0.2*	*56.5*	*278.8*	*Long*	*Short*
T11	0.1	63.4	537.6	Short	Short	T01	0.2	87.4	374.5	Short	Short
T05	0.0*	56.6	1446.4	Short	Short	T05	0.1	68.8	523.1	Short	Short
T10	0.0*	56.5	8506.6	Short	Short	T10	0.0*	70.0	8150.6	Short	Short

Digital biomarker (DBM) area refers to percent of total tumor area classified as a biomarker. Outcome refers to the actual PFI group. Italicized rows are slides incorrectly classified by the neural network.

*Does not represent true 0%.

We subsequently classified the validation test set (22 slides, 22 women) into short and long PFI groups (Table 3). Classification was very similar for both replicates of the neural network. Given that the absolute area classified as either long or short is not comparable between slides, the classifier decision is based on the relative percent area ratio of short to long PFI features (short/long) in rank order, with a cutoff of 30 providing the best fit. Replicate 2 provided a higher specificity than replicate 1; all slides misclassified by replicate 2 were also misclassified in replicate 1. Applying replicate 2 to the test set: 8 of 11 short PFI group samples and 10 of 11 long PFI group samples were correctly classified, for a sensitivity of 73% and a specificity of 91%. The positive predictive value is 89%, with an overall accuracy of 82%.

In order to determine if the neural network results were due to a retrospectively apparent similarity between slides (either morphologic or artifactual), the three highest-scoring test set WSI (as per Table 3) for each PFI category were visualized and compared. No obvious morphologic or artifactual parallels were observed by reviewing the test set WSI visual results alongside the classification result data in Table 3. The three slides with the lowest ratio of short to long PFI-associated features all had SET-predominant morphology; regions identified as PFI-L-associated tended to have solid architecture. Of the three slides with the highest ratio of short to long PFI-associated features, two were predominantly SET, with one showing uniform endometrioid architecture. In these slides, large tumor areas (70–87%) were identified as PFI-S-associated and consisted of variable morphologies: solid, endometrioid, papillary. Representative images are presented in the supplementary information (Supplementary Fig. S1).

## Discussion

This proof-of-concept study provides evidence that a deep learning model can use H&E histology alone to predict the biological response of high-grade serous carcinoma to adjuvant platinum chemotherapy, using PFI as a proxy. Our results provide evidence that a weakly supervised convolutional neural network can discriminate extremes in patient outcome with high specificity. This finding is of both practical and conceptual importance, as there are currently no validated tissue-based prognostic or predictive markers for primary platinum-based treatment in use for HGSC^30^. Surprisingly, and in contrast to many other tumor types, pathologist evaluation of HGSC tumor tissue provides almost no predictive or prognostic information beyond the diagnosis itself. This work is predicated in the belief that as-of-yet unidentified clinically relevant information exists within H&E slides of tumor.

We utilized a well-curated patient cohort and selected the extremes of patient response to initial therapy in order to maximize the potential differences between the two groups of patients in this initial work. The specific time periods (≤ 6 months, ≥ 18 months) were chosen to roughly align with standard clinical definitions of platinum refractory/resistance and platinum sensitive, respectively, and to capture the small group of patients with long term survival^31^. Given that the patient cohort is so tightly stratified by clinical presentation and treatment type, PFI is highly likely to be a relevant reflection of the biological behavior of these tumors. The carefully curated cohort is critical to our study design, as we know that a variety of clinical factors, such as complete macroscopic cytoreduction (R0 vs > R0), are associated with significant outcome differences. The ability to select carefully stratified groups of patients with different outcomes is required to isolate and focus the results on the most relevant tissue findings.

We hypothesized that the most relevant prognostic information would be found within the tumors themselves; therefore we focused our annotations and subsequent image analysis only on tumor regions and left the background tissue unexplored. Specific morphologic features have been described in HGSC, most notably that in tumors with BRCA1 and other HR pathway mutations, solid, pseudoendometrioid, and transitional (SET) patterns, rather than the classic micropapillary architecture, are more often identified^19,20^. However, these patterns have not been sufficiently discriminatory to predict mutation status or treatment response. Overall, we noted that predominantly papillary tumors were less common than predominantly SET/ambiguous pattern tumors, and that tumors with a predominantly papillary architecture were evenly distributed between the short and long PFI groups (Table 2) in both our training and test sets. The BRCA status is unknown in nearly all cases, as it was not being performed clinically at the time of diagnosis; the two cases with known germline BRCA mutation status are both negative, and both showed predominantly papillary architecture. Similarly to previous reports, the presence of tumors with uniform morphology is uncommon, and predominantly identified in the non-classic SET/ambiguous group^20^. Our findings expand the concept of a morphological/functional relationship by providing an explicit mechanism for incorporating tumor heterogeneity and ambiguity into morphologic evaluation, via the identification of quantifiable digital biomarkers for predicting outcome. Our model has the potential to serve as a foundation for the development of validated systems that would provide additional prospective information to guide patients at the start of their treatment.

Replicate trainings of the final neural network produced very similar classifications results (Table 3) and identified similar-appearing digital biomarkers (high confidence pixel areas). This finding is reassuring, and provides support for our proposition that the findings produced by this neural network are real, rather than random variations identified within the tumor histology. While overfitting should always be considered, our overall accuracy of > 80% on the independent test set indicates sufficient generalizability. The Aiforia platform does not currently provide for systematic review of our visual results, so the depicted morphology must be interpreted with caution. That said, the digital biomarker morphology (as shown in Fig. 2) appears to provide some hints; the findings for PFI-L tend to be more solid and pleomorphic, while those for PFI-S tend to have more papillary features and appear more variable overall. Future directions include closer study of these digital biomarkers to examine which features are most important, and to investigate what they represent. This focused study of the tumor cells within this extremely heterogeneous disease will direct new questions toward the underpinnings of HGSC treatment response and progression, and we believe that looking more closely at restricted regions of tumor tissue regions will prove valuable. Others have also shown that neural networks have the potential to address the role of tumor morphology in serous carcinoma^32^; our work considers this question purposefully by specifically addressing only high grade serous carcinoma, and using a patient cohort that has been appropriately verified and stratified for an up-to-date diagnosis, staging, and PFI status.

It is interesting to note that within these remarkably heterogenous tumors, digital biomarkers for PFI-S predominate and were identified in all WSI. Relatedly, almost all WSI had at least focal PFI-L-associated regions. Though these findings must be interpreted with caution as the results are directly related to the gain used to visualize the output, they are intriguing. The idea that all tumors have “good outcome” and “poor outcome” regions is consistent with the concept of genetic heterogeneity in serous carcinoma, and this technique has the potential to provide a mechanism for assessing the relative proportions quickly and in a clinically relevant manner. In the future, these findings provide a solid foundation for investigation via spatial transcriptomic techniques to identify and confirm differences in gene expression between the digital biomarker regions, and especially to investigate differences between the PFI-L and PFI-S digital biomarkers.

There are several limitations to this pilot study. First, the sample size is small; verification of the accuracy of the model will need testing on a much larger sample size, and an external patient cohort. Second, molecular testing of ovarian tumors was not routinely performed on clinical samples in Finland during the time these samples were obtained, and therefore this information is lacking. While this is not critical to the performance of the classifier, a clinical tool would require this additional information to be most useful. Third, our samples were restricted to adnexal tumors, leaving omental tumor morphology unexplored and potentially limiting the neural network generalizability. Fourth, although the methods used in the study are not black-box as users can always verify neural networks results visually in Aiforia, deeper understanding of the histological features associated with the outcomes requires further studies. Despite these limitations, this work provides substantial evidence that a neural network is able to provide clinically relevant additional prognostic information to guide the prospective care of patients with HGSC carcinoma.

Our findings are in keeping with the emerging body of literature in other cancer types^26,27,33^, indicating that neural networks are able to extract additional clinically relevant information from routine H&E slides. Our study is unique among the literature in the sense that sophisticated prognostication and histomorphology-based predictive and prognostic biomarkers are not currently available to HGSC patients. This work shows, for the first time, that deep learning neural networks can discriminate extremes in patient outcome in high grade extrauterine serous carcinoma using tumor histology alone.

## Supplementary Information

Supplementary Information.

## Data Availability

The data that support the findings of this study are available from the Helsinki Biobank but restrictions apply to the availability of these data, which were used with permission for the current study, and are not publicly available. Data are however available from the authors upon reasonable request and with permission. The image analysis data and the AI model’s results are available at cloud.aiforia.com upon reasonable request and with permission.
