# Supplementary material for: Artificial intelligence-based image analysis can predict outcome in high-grade serous carcinoma via histology alone
Source: Sci Rep. 2021 Sep 27;11:19165. doi: 10.1038/s41598-021-98480-0 (PMC8476598; doi:10.1038/s41598-021-98480-0)

Supplementary Information.

Artificial intelligence-based image analysis can predict outcome in high-grade serous carcinoma via histology alone.

Anna Ray Laury[1]*, Sami Blom[4], Tuomas Ropponen[4], Anni Virtanen[3], Olli Mikael Carpén[1,2,3]

Supplementary Table S1. Training parameters in the Aiforia platform.

	Neural Net 1	Neural Net 2	Neural Net 3
Neural architecture	Ultra Complex	Ultra Complex	Extra Complex
Field of view (µm)	250	250	200
Training parameters			
Weight decay	0.0001	0.0001	0.0001
Mini-batch size	5	5	20
Mini-batches per iteration	20	20	20
Iterations without progress	500	500	2000
Initial learning rate	0.1	0.1	0.1
A priori rate	0.5	0.5	0.5
Minimum training window size	256px	256px	256px
Maximum training window size	2048px	2048px	2048px
Image augmentation			
Scale	-1/1.01	-1/1.01	-1/1.01
Aspect Ratio	1	1	1
Maximum shear	1	1	1
Luminance (min/max)	-1/1	-1/1	-1/1
Contrast (min/max)	-1/1.01	-1/1.01	-1/1.01
Maximum white balance change	1	1	1
Noise	0	0	2

Supplementary Figure S1.

Neural network-identified morphologic regions within the test set WSI. (a) Tumor regions identified as associated with PFI-S (digital biomarkers). Representative images from the three highest scoring slides (highest ratio of short/long-associated tumor area) identified by the neural network on the WSI (L to R; T1,T5,T10). (b) Tumor regions identified as associated with PFI-L (digital biomarkers). Representative images from the three highest scoring slides (lowest ratio of short/long-associated tumor area) identified by the neural network on the WSI (L to R; T7,T15,T16). Tile size is 4000x4000px.

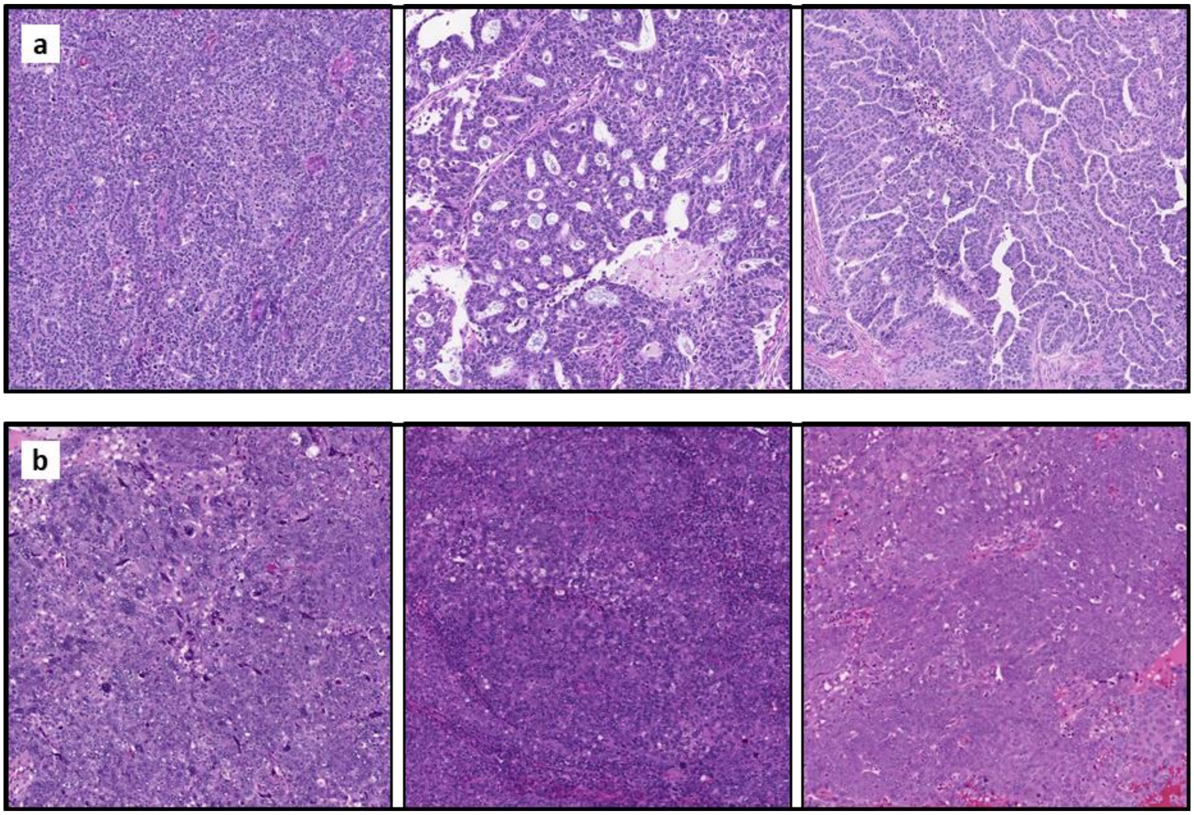

Supplement: Supplementary file 1 — Supplementary Information. [file 41598_2021_98480_MOESM1_ESM.pdf]
